# Chinese Herbal Injections for Primary Nephrotic Syndrome in Adults: A Systematic Review and Network Meta-Analysis

**DOI:** 10.1155/2020/1047489

**Published:** 2020-02-24

**Authors:** Shisheng Han, Tianwen Yao, Yanhua Lu, Yan Lu, Yanqiu Xu, Yi Wang

**Affiliations:** ^1^Department of Nephrology, Yueyang Hospital of Integrated Traditional Chinese and Western Medicine, Shanghai University of Traditional Chinese Medicine, Shanghai 200437, China; ^2^Department of Internal Medicine, Youyi Street Community Health Service Center of Baoshan District, Shanghai 201900, China

## Abstract

Primary nephrotic syndrome (PNS) is a common renal disease that presents with heavy proteinuria and hypoalbuminemia. Despite notable advances in its treatment, some patients show poor responses and clinical outcomes when treated with conventional Western medicine (WM). Chinese herbal injections (CHIs) have been reported to have beneficial effects for PNS. The aim of the present study was to comprehensively determine the efficacy and safety of CHIs for PNS in adults using a network meta-analysis approach. PubMed, Embase, the Cochrane library, and four Chinese databases were systematically searched to identify randomized controlled trials (RCTs) using CHIs for treatment of PNS published before June 1, 2019. Quality assessment of the identified RCTs was performed according to the Cochrane Handbook. Pooled odds ratios (OR) or mean differences (MD) with corresponding 95% confidence intervals (CI) were calculated for discrete or continuous variables, respectively. The primary outcome was complete/total remission and secondary outcomes were serum albumin and urinary protein excretion. The surface under the cumulative ranking curve (SUCRA) value and cluster analyses were used to rank treatment by probability. Eighty-five studies involving 11 CHIs and 5801 subjects were included. Compared with WM alone, CHI plus WM showed an improved complete/total remission rate as well as higher serum albumin and lower 24-hour urinary protein excretion, except in the following: Yinxingye injection plus WM did not improve the total remission rate, and Dengzhanhua or Xueshuantong injection plus WM did not lower the 24-hour urinary protein excretion. Either Danhong (DH) or Dengzhanhua (DZH) injection plus WM was the preferable treatment for PNS based on SUCRA and cluster analyses of clinical remission and adverse events. However, considering that literature in this area is limited, these results need further validation. CHIs administered as adjuvants to WM showed favourable outcomes for PNS. DH + WM and DZH + WM might be the potential optimal therapies for PNS.

## 1. Introduction

Primary nephrotic syndrome (PNS) is a prevalent glomerular disease characterised by excessive proteinuria and hypoalbuminemia, manifesting with histological differences, such as minimal change disease and membranous nephropathy [1]. Renin-angiotensin system (RAS) blockade and/or immunosuppressive agents are recommended treatments. However, patients show varying responses; 20%–50% of patients continue with substantial proteinuria, which is associated with higher risk of end stage kidney disease (ESKD) [2–4]. Additionally, failure of clinical remission is associated with increased risk of various complications, such as infections and thromboembolisms. Moreover, recommended treatments for PNS can also cause unwanted side effects [5–7]. Traditional Chinese medicine (TCM) has been documented to have a beneficial effect for PNS diagnosed based on individual symptoms, but not the pathological type; thus, TCM might be a potential adjuvant or alternative treatment for PNS [8–10]. Chinese herbal injections (CHIs) are innovative formulations of herbs with high bioavailability and rapid action that are widely administered in China [11]. Several traditional pairwise meta-analyses have suggested that CHIs might be effective treatments for PNS [12, 13]. However, since pairwise meta-analyses only directly compare two interventions, a comparison of the therapeutic effects of many different CHIs for PNS has not been performed. Network meta-analysis (NMA) can give a unified, coherent analysis of direct and indirect evidence as well as rank the probability of optimal treatment. Therefore, in this study, NMA was used to determine the relative efficacy and safety of different CHIs for PNS and predict the best candidate treatment.

## 2. Materials and Methods

### 2.1. Compliance with Ethical Standards

This work was performed according to the PRISMA extension statement for network meta-analyses ([Supplementary-material supplementary-material-1]). The protocol of the present NMA was registered in PROSPERO: CRD42019133746. Because all the data were based on previously published literature, ethical approval and informed consent were not applicable.

### 2.2. Literature Searching

PubMed, Embase, Cochrane Central Register of Controlled Trials (CENTRAL), and four Chinese databases (the China National Knowledge Infrastructure (CNKI) database, WangFang database, China Science and Technology Journal (VIP) database, and China Biology Medicine (CBM) database) were searched for eligible studies published prior to June 1, 2019. Medical subject headings and free-text searches were used by combining the following three domains without language restrictions: CHI, nephrotic syndrome, and randomized controlled trial (RCT). The specific search terms are shown in [Supplementary-material supplementary-material-1].

### 2.3. Inclusion and Exclusion Criteria

The inclusion criteria were identified by the Participants, Interventions, Comparisons, Outcomes, and Study design (PICOS) framework: (1) participants: adults diagnosed with PNS; (2) interventions and comparisons: the experimental group was administered a CHI or CHI plus conventional Western medicine ((WM) defined as RAS inhibitors, steroids, immunosuppressive agents, and symptomatic treatments that were the same in both groups), and the control group was treated with WM alone or another CHI with/without WM; (3) outcomes: the primary outcomes were complete remission (CR) or total remission (TR), which were assessed according to the definition provided in each study; secondary outcomes were 24-hour urinary protein excretion, serum albumin, total cholesterol (TC), triglycerides, serum creatinine, adverse reactions (ADRs), ESKD, and all-cause mortality; and (4) study design: RCT.

The exclusion criteria were as follows: (1) children with nephrotic syndrome (NS); (2) no data available for analysis; or (3) secondary NS.

### 2.4. Data Extraction and Quality Assessment

We extracted the following data from each included study: first author; publication year; location; baseline information for all groups (sample size, age, and gender); diagnostic criteria for PNS; details of intervention and control; duration of follow-up; outcomes; and sources of publication. The Cochrane Handbook assessment tool for risk of bias (version 5.1.0) was used to assess the quality of each trial. Data extraction and quality assessment were independently performed by two investigators each, and disagreements were solved by a third reviewer.

### 2.5. Statistical Analysis

All statistical analyses were performed with STATA software (version 14.0) using the network command [14]. Pooled odds ratios (OR) or mean differences (MD) with 95% confidence intervals (CIs) were calculated for discrete or continuous data, respectively. The random-effects network meta-analysis was conducted to compare all classes of CHIs for each prespecified outcome under the frequentist framework. Inconsistency tests were performed to explore the network heterogeneity between designs; *P* < 0.05 was considered to be significant heterogeneity. The loop-specific approach was used to compare the difference between direct and indirect comparisons within triangular loops in a network; random-effects pairwise meta-analyses were performed and presented as main results if significant inconsistency existed. The surface under the cumulative ranking curve (SUCRA) value was calculated to rank the interventions. Cluster analysis was utilized to comprehensively compare the efficacy and safety of interventions. Publication bias was tested by the comparison-adjusted funnel plot, Begg's test, and Egger's test. We performed sensitivity analyses by comparing the results between random- and fixed-effects models.

## 3. Results

### 3.1. Characteristics of Eligible Studies

**Figure 1:**
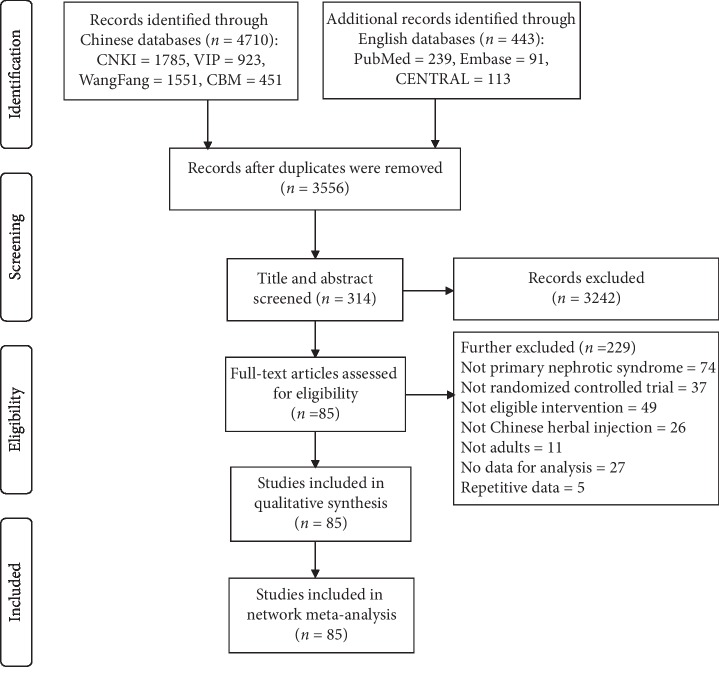
Flow diagram of literature selection. n, number of publications; CNKI, China National Knowledge Infrastructure database; VIP, China Science and Technology Journal database; WangFang, WangFang database; CBM, China Biology Medicine database; CENTRAL, Cochrane Central Register of Controlled Trials.

**Figure 2:**
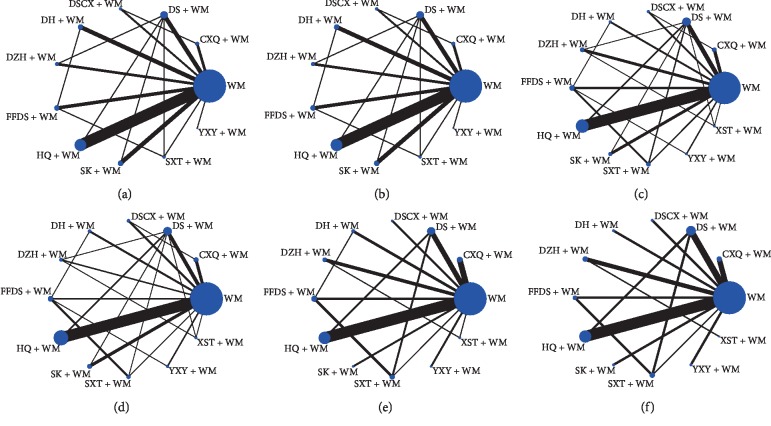
Network graphs for each outcome. (a) Complete remission; (b) total remission; (c) 24-hour urinary protein excretion; (d) serum albumin; (e) total cholesterol; (f) triglycerides.

A total of 3556 publications were retrieved from electronic databases after removing duplications. Eighty-five studies were identified for meta-analyses according to the inclusion and exclusion criteria. Details of the literature selection process are shown in Figure 1. All trials were conducted in China and published in Chinese. Eleven CHIs were reported in these RCTs: Chuanxiongqin (CXQ), Danshen (DS), Danshenchuanxiongqin (DSCX), Danhong (DH), Dengzhanhua (DZH), Fufangdanshen (FFDS), Huangqi (HQ), Shenkang (SK), Shuxuetong (SXT), Yinxingye (YXY), and Xueshuantong (XST) injection. There were 5801 participants (3331 males and 2470 females) enrolled in the 85 RCTs. All experimental groups (3155 participants) received CHIs plus WM and control groups (2967 participants) received WM alone or another CHI with WM. Characteristics of the selected studies are depicted in [Supplementary-material supplementary-material-1]. The network graphs for each outcome are presented in Figure 2.

### 3.2. Risk of Bias Assessment

**Figure 3:**
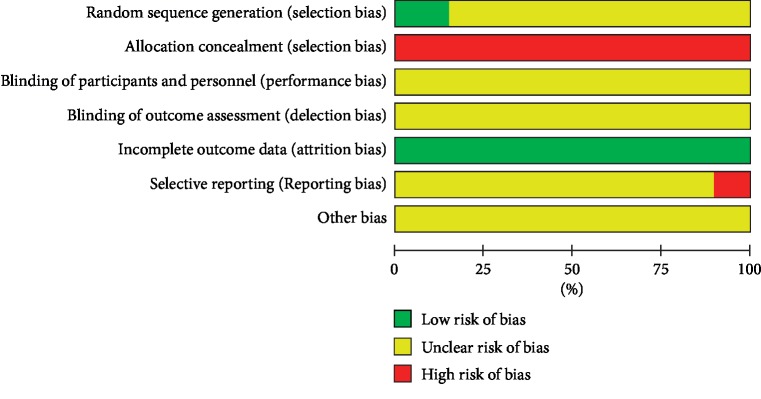
Summary of the risk of bias. The risk of bias assessment revealed the RCTs to be of poor methodological quality.

Thirteen of the 85 studies described appropriate methods for generating random sequences; thus, their selection bias risk was considered low. The remaining studies were classified as unclear risk because they only mentioned “random” selection. None of the studies reported the processes used for allocation concealment or blinding. Thus, allocation concealment was considered as high risk. Performance bias and detection bias were classified as unclear risk due to insufficient information. All studies had complete data, so the attrition bias was evaluated as low risk. Nine studies did not report all the outcomes described in the methods section, so their reporting bias was deemed high risk. The other biases were classified as unclear risk because there were no available details on which to evaluate the risk of bias. In summary, the quality of included RCTs was poor (Figure 3).

### 3.3. CR and TR

Fifty studies, including 10 CHIs, reported CR and TR outcomes in patients with PNS. NMA results indicated that CXQ + WM, DS + WM, DSCX + WM, DH + WM, DZH + WM, FFDS + WM, HQ + WM, SK + WM, SXT + WM, and YXY + WM each significantly improved CR rate in PNS patients compared to WM alone (OR, 1.81–3.64). In addition, SXT + WM had a significantly higher CR rate than FFDS + WM (OR, 2.02; 95% CI, 1.13–3.61), and FFDS + WM had a significantly lower CR rate than DH + WM (OR, 0.53; 95% CI, 0.32–0.86).

NMA results showed that all the groups of PNS patients receiving CHI + WM had significantly better TR rates than those receiving WM alone (OR, 2.92–6.54), except for the YXY + WM group. SXT + WM had a significantly higher TR rate than FFDS + WM (OR, 2.65; 95% CI, 1.33–5.28), and FFDS + WM had a significantly lower TR rate than DZH + WM (OR, 0.35; 95% CI, 0.13–0.98) and DH + WM (OR, 0.50; 95% CI, 0.27–0.91) (Table 1).

### 3.4. Serum Albumin and Urinary Protein Excretion

Sixty-one studies, involving 11 CHIs, reported serum albumin levels and 73 publications reported 24-hour urinary protein excretion. NMA showed that all the groups of PNS patients receiving CHIs + WM (CXQ + WM, DS + WM, DSCX + WM, DH + WM, DZH + WM, FFDS + WM, HQ + WM, SK + WM, SXT + WM, YXY + WM, and XST + WM) had higher serum albumin levels than groups receiving WM alone (MD, 3.00–5.98). When compared to the group receiving CXQ + WM, the groups receiving SXT + WM (MD, 2.97; 95% CI, 0.05–5.90), SK + WM (MD, 2.91; 95% CI, 0.53–5.28), or DH + WM (MD, 2.82; 95% CI, 0.10–5.53) showed significantly higher serum albumin levels. Additionally, groups of PNS patients receiving SXT + WM (MD, 2.94; 95% CI, 0.34–5.53), SK + WM (MD, 2.87; 95% CI, 0.96–4.79), HQ + WM (MD, 1.63; 95% CI, 0.12–3.14), or DH + WM (MD, 2.78; 95% CI, 0.33–5.24) had significantly higher serum albumin levels than the group receiving DS + WM (Table 2).

Due to inconsistency in the urinary protein excretion data (*P*=0.01), the pairwise meta-analyses are shown as main results. The results suggest that all groups of PNS patients receiving CHIs + WM had significantly lower urinary protein than groups receiving WM alone (MD,−0.70 to −1.92), except for the groups receiving XST + WM or DZH + WM. The group receiving SXT + WM had significantly reduced proteinuria compared to those receiving FFDS + WM (MD,−1.28; 95% CI,−2.01 to −0.55) or DS + WM (MD,−1.93; 95% CI,−2.30 to −1.56). The group receiving SK + WM showed significantly lower urinary protein excretion than those receiving DS + WM (MD,−1.70; 95% CI,−2.58 to −0.82). The group receiving DSCX + WM had significantly decreased urinary protein excretion than those receiving CXQ + WM (MD,−0.53; 95% CI,−0.77 to −0.30). The group receiving FFDS + WM showed significantly higher urinary protein excretion than those receiving DH + WM (MD, 1.06; 95% CI, 0.43–1.70) (Table 2).

### 3.5. TC and Triglycerides

Fifty-two and 42 RCTs, including 11 CHIs, reported TC and triglycerides, respectively. Because of the inconsistency of the data (*P*=0.01), results for TC were calculated using pairwise meta-analysis. Groups of PNS patients receiving YXY + WM, HQ + WM, FFDS + WM, DZH + WM, DS + WM, or CXQ + WM had significantly lower TC than those receiving WM alone. The NMA results for triglycerides indicated that seven CHI + WM groups (SXT + WM, SK + WM, HQ + WM, FFDS + WM, DZH + WM, DS + WM, and CXQ + WM) had significantly lower triglycerides than groups receiving WM alone ([Supplementary-material supplementary-material-1]).

### 3.6. ADR and Other Outcomes

Twenty-three studies addressed ADRs; 11 RCTs clearly documented that there were no ADRs in either group. The details of ADRs are shown in Table 3; overall, no serious adverse events were reported. We next performed NMA for total ADR and found there were no significant differences between any of the groups, except the YXY + WM group, which showed lower ADR than the CXQ + WM group ([Supplementary-material supplementary-material-1]). There were no significant differences between serum creatinine levels in any of the groups ([Supplementary-material supplementary-material-1]). None of the studies reported on the incidence of ESKD or all-cause mortality.

### 3.7. Rank Probability Based on SUCRA and Cluster Analysis

**Figure 4:**
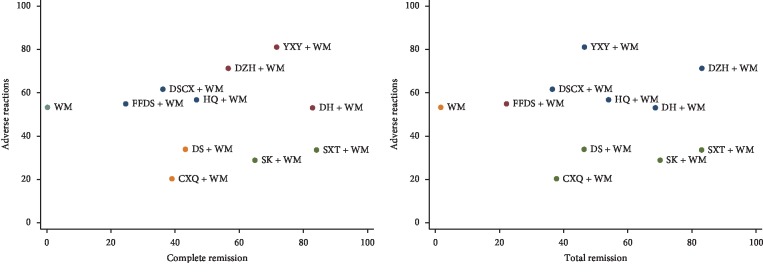
Cluster analyses based on complete remission, total remission, and adverse reaction. DH + WM and DZH + WM showed preferable outcomes.

The rank of interventions for each outcome, based on SUCRA, is presented in Table 4. Compared to WM alone, combining WM with CHIs showed better clinical benefits. Cluster analyses showed that DH + WM and DZH + WM might be the preferred interventions for PNS based on the outcomes of efficacy and safety (CR, TR, and ADR) (Figure 4). Moreover, SK + WM, SXT + WM, and YXY + WM also showed preferred efficacy outcomes; however, this should be interpreted with caution due to the low number of studies reporting results with YXY + WM and SXT + WM (1 and 3, respectively).

### 3.8. Inconsistency Test

Inconsistency tests suggested that direct and indirect comparisons were consistent for the outcomes of CR, TR, serum albumin, triglycerides, and serum creatinine. However, several loops showed inconsistency for the outcomes of TC and urinary protein excretion ([Supplementary-material supplementary-material-1]), so pairwise meta-analyses were used as the main results for these two outcomes.

### 3.9. Publication Bias

**Figure 5:**
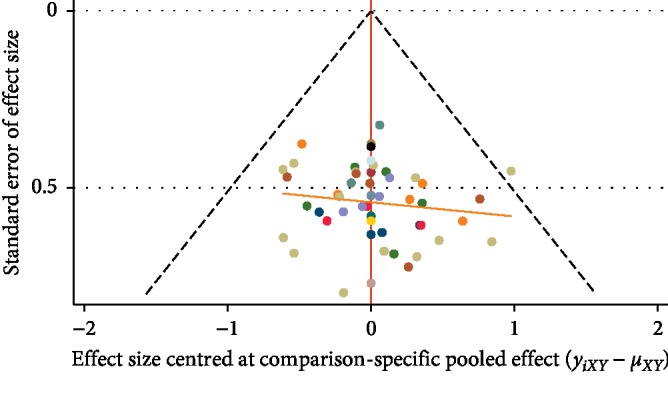
Comparison adjusted funnel plot for complete remission.

The comparison-adjusted funnel plot was used to evaluate publication bias according to the primary outcome of CR. Because the funnel plot was relatively symmetrical (Figure 5), we next used Begg's test and Egger's test to check the publication bias quantitatively, which both showed no statistical differences (Begg's test, *z* = 0.80, *P*=0.42; Egger's test, *t* = 1.74, *P*=0.09).

### 3.10. Sensitivity Analysis

We performed sensitivity analysis of the primary outcome of clinical remission by comparing the results of random-effects models with fixed-effects models. The sensitivity analysis showed a stable result.

## 4. Discussion

Although many new immunological treatments, such as rituximab, have been used in PNS, some patients showed unsatisfactory responses and subsequent unfavourable clinical outcomes [15, 16]. An increasing amount of evidence indicates that TCM herbal formulations, because of their diversified effects, might be an effective complementary or alternative regimen for chronic kidney disease, especially primary glomerular disease [17]. Injection of herbal formulations is widely prescribed in China, especially for hospitalised patients; however, the efficacy and safety of these herbal injections are not clear.

This is the first NMA based on RCTs to assess the efficacy and safety of CHIs for treating PNS in adults. In the present NMA, the use of eleven CHIs as adjuvants to WM in PNS was comprehensively compared based on the outcomes of clinical remission, urinary protein excretion, serum albumin, TC, triglycerides, serum creatinine, and ADRs. The treatments evaluated in this study were CXQ + WM, DS + WM, DSCX + WM, DH + WM, DZH + WM, FFDS + WM, HQ + WM, SK + WM, SXT + WM, YXY + WM, and XST + WM. The results suggested that CHIs plus conventional Western pharmaceutical agents are associated with significantly better measured PNS outcomes than WM alone (the effect on serum creatinine was not significantly different between the groups). Among the treatments including CHIs, DH + WM and DZH + WM might be the preferable therapies, according to the results of SUCRA and cluster analyses.

According to TCM theory, the herbs used to formulate the included injections identified in this study are blood-activating and stasis-removing drugs, with the exception of Huangqi (Astragali Radix), which is a Qi-invigorating herb. This indicates the importance of blood stasis syndrome and Qi deficiency syndrome in PNS, which is consistent with previous studies [18]. According to TCM theory, Qi deficiency is involved in the pathogenesis of nephrotic syndrome, and blood stasis acts as a key pathological factor to further complicate the condition [8]. A TCM syndrome study in PNS showed that Qi deficiency is one of the most common TCM syndrome types, whose score correlated significantly with higher urine protein/creatinine ratio and TC level [19]. Moreover, 91.19% of patients with primary glomerular disease were diagnosed as blood stasis syndrome, of which PNS had a higher syndrome score [20]. Additionally, the blood stasis syndrome score was positively correlated with urine protein ration, triglyceride, and cholesterol. The results also indicated that the more severe the proliferative and sclerotic renal pathologies were, the higher the score was [21]. Similar results were found in IgA nephropathy [22]. In this context, the crucial TCM concept for treating PNS is to invigorate Qi, activate blood, and remove stasis, which might increase the rationality and applicability of our results. Idiopathic membranous nephropathy, which, besides IgA nephropathy, is a prevalent pathological type of PNS in China [23], has been shown to positively respond to the Qi-invigorating and stasis-removing method; moreover, the most frequently used Chinese medicines have been reported to be Astragali Radix, Angelicae Sinensis Radix, Chuanxiong Rhizoma, and Salvia Miltiorrhiza [24].

Previous studies have suggested that the preferred CHIs have positive effects on glomerular disease, such as reducing proteinuria and blood lipid. In addition, the therapeutic mechanisms for these effects have been partly revealed through experimental studies. For example, several pairwise meta-analyses have shown the beneficial effects of DZH (Breviscapus) as an antiproteinuria, in improving albumin level, and in lowering cholesterol and triglyceride for hypertension-induced renal injury and diabetic nephropathy; the mechanism may be correlated with synergetic suppression of increased oxidative stress [25–27]. Recent studies have suggested that DH can effectively decrease urine protein excretion by multiple pathways, as well as confer lipid-lowering effects [28–30]. DH is a herbal formulation of Danshen (Salvia Miltiorrhiza) and Honghua (Carthami Flos). Their main components, such as salvianolic acid, have been shown to have a podocyte-protection effect in an adriamycin-induced nephrotic syndrome rat model and a mouse podocyte injury cell model, which may be one of the mechanisms of DH's renoprotective effects [31]. In the present study, SK and SXT also showed preferred efficacy outcomes. SK is a Chinese medical-standardised product extracted from Dahuang (Rhei Radix), Huangqi (Astragali Radix), Danshen (Salvia Miltiorrhiza), and Honghua (Carthami Flos), formulated according to TCM theory and widely used in the clinical treatment of kidney disease. Either the injection of SK or its components have been found to alleviate glomerular damage via antioxidation and inhibit proliferation of renal mesangial cells [32, 33]. SXT is extracted from Shuizhi (Hirudo) and Dilong (Pheretima), which are powerful blood-activating and stasis-removing herbs in TCM theory. Pharmacological studies have shown that Shuizhi plays a role in anticoagulation, preventing platelet aggregation and improving hemorheological parameters, which might reflect the mechanism through which SXT is beneficial for PNS [34]. In addition, the Dilong has been shown to reduce glomerular injury and urinary albumin excretion, possibly via suppression of mesangial matrix expansion and activation of matrix metalloproteinase-2 [35].

Apart from efficacy, the safety of CHI in the treatment of PNS is an important issue. Although CHIs, including those evaluated in this study (such as Danshen, Xueshuantong, and Shuxuetong injection), have been used in China for more than half a century, research assessing their safety should be systematically conducted because of their higher risk of adverse effects compared to other forms of TCM medications and the higher percentage of new serious adverse events compared to conventional injections [36]. Few studies included in this NMA focused on adverse reactions; therefore, more evidence is needed to verify the safety of these CHIs.

Potential limitations should be considered when interpreting the results of this study. First, significant heterogeneity and inconsistency were observed when performing comparisons for urinary protein excretion and cholesterol; therefore, we could only conduct pairwise meta-analyses for these outcomes and rank results could not be obtained. Second, the enrolled population was exclusively Chinese; thus, whether our results are applicable to a more general population remains unknown. Third, although all patients were diagnosed as having PNS with the same criteria, the pathological types varied, which may have caused potential heterogeneity. Fourth, the different dosage and duration for CHIs may have led to potential bias. Because the methodological quality of the included RCTs was low and few studies reported adverse events, further high-quality RCTs are required to validate the efficacy and safety of CHIs. We also propose some suggestions towards RCTs on CHI for the treatment of PNS. First, RCTs should be registered in advance to ensure the transparency of the trial process. Second, researchers should report the trial process in as much detail as possible according to the RCT reporting standard, in order to improve the quality, credibility, applicability, and value of the literature. Regarding its design and contents, the present study has two particular strengths. First, it conducted a comprehensive literature search and a comparison for eleven CHIs in treating PNS. Second, an eligibility criterion was formulated before the NMA to reduce the clinical heterogeneity as far as possible. Finally, the results demonstrated the beneficial effects in multiple aspects and provided several clinical suggestions for treatment of PNS.

## 5. Conclusions

Taken together, the current work demonstrates that adjuvant therapy with CHIs could have favourable clinical outcomes for PNS; DH + WM and DZH + WM might be the potential optimal PNS treatments. Due to the limited evidence, high-quality studies with reasonable design are needed to confirm the efficacy of CHI. Moreover, their safety also requires a systematical assessment.

## Figures and Tables

**Table 1:** Network meta-analyses of complete remission and total remission.

										Total remission
YXY + WM	0.52 (0.05, 5.70)	0.67 (0.06, 7.03)	0.82 (0.08, 8.48)	1.37 (0.13, 14.56)	0.48 (0.04, 5.68)	0.68 (0.06, 7.15)	1.07 (0.10, 11.55)	0.90 (0.09, 9.49)	1.08 (0.09, 12.43)	3.15 (0.31, 31.62)
0.89 (0.31, 2.60)	SXT + WM	1.29 (0.57, 2.92)	1.59 (0.73, 3.45)	**2.65 (1.33, 5.28)**	0.94 (0.31, 2.81)	1.31 (0.59, 2.91)	2.08 (0.84, 5.10)	1.75 (0.83, 3.70)	2.10 (0.73, 6.01)	**6.12 (3.10, 12.08)**
1.18 (0.44, 3.15)	1.33 (0.65, 2.69)	SK + WM	1.23 (0.67, 2.28)	2.05 (1.00, 4.22)	0.72 (0.27, 1.95)	1.02 (0.50, 2.05)	1.61 (0.75, 3.47)	1.36 (0.77, 2.39)	1.63 (0.63, 4.17)	**4.74 (2.89, 7.77)**
1.41 (0.55, 3.59)	1.58 (0.83, 3.02)	1.19 (0.73, 1.93)	HQ + WM	1.66 (0.86, 3.20)	0.59 (0.23, 1.52)	0.82 (0.44, 1.54)	1.30 (0.65, 2.62)	1.10 (0.62, 1.96)	1.32 (0.54, 3.20)	**3.84 (2.64, 5.59)**
1.80 (0.67, 4.81)	**2.02 (1.13, 3.61)**	1.52 (0.86, 2.70)	1.28 (0.78, 2.09)	FFDS + WM	**0.35 (0.13, 0.98)**	**0.50 (0.27, 0.91)**	0.78 (0.35, 1.74)	0.66 (0.34, 1.31)	0.79 (0.30, 2.08)	**2.31 (1.35, 3.95)**
1.27 (0.43, 3.74)	1.42 (0.61, 3.30)	1.07 (0.52, 2.21)	0.90 (0.46, 1.75)	0.70 (0.34, 1.46)	DZH + WM	1.40 (0.51, 3.84)	2.22 (0.77, 6.37)	1.88 (0.73, 4.82)	2.24 (0.68, 7.36)	**6.54 (2.72, 15.71)**
0.95 (0.36, 2.52)	1.06 (0.54, 2.09)	0.80 (0.45, 1.41)	0.67 (0.41, 1.09)	**0.53 (0.32, 0.86)**	0.75 (0.36, 1.54)	DH + WM	1.58 (0.73, 3.42)	1.34 (0.68, 2.61)	1.60 (0.62, 4.12)	**4.66 (2.83, 7.68)**
1.59 (0.57, 4.41)	1.78 (0.83, 3.84)	1.35 (0.71, 2.54)	1.13 (0.65, 1.98)	0.88 (0.47, 1.68)	1.25 (0.58, 2.74)	1.68 (0.89, 3.16)	DSCX + WM	0.85 (0.40, 1.78)	1.01 (0.43, 2.37)	**2.95 (1.64, 5.31)**
1.46 (0.56, 3.81)	1.64 (0.86, 3.12)	1.23 (0.76, 1.99)	1.04 (0.68, 1.59)	0.81 (0.48, 1.37)	1.15 (0.59, 2.25)	1.54 (0.91, 2.61)	0.92 (0.50, 1.67)	DS + WM	1.20 (0.47, 3.02)	**3.49 (2.21, 5.51)**
1.57 (0.53, 4.58)	1.76 (0.76, 4.06)	1.32 (0.64, 2.72)	1.11 (0.58, 2.14)	0.87 (0.42, 1.79)	1.23 (0.53, 2.89)	1.65 (0.81, 3.39)	0.98 (0.50, 1.95)	1.07 (0.54, 2.14)	CXQ + WM	**2.92 (1.31, 6.51)**
**3.25 (1.33, 7.95)**	**3.64 (2.02, 6.57)**	**2.75 (1.84, 4.11)**	**2.31 (1.76, 3.04)**	**1.81 (1.20, 2.72)**	**2.56 (1.40, 4.70)**	**3.43 (2.30, 5.13)**	**2.04 (1.25, 3.33)**	**2.23 (1.57, 3.15)**	**2.08 (1.14, 3.77)**	WM

Complete remission										

The results of network meta-analyses are presented as odds ratios (95% confidence intervals) for complete remission (bottom left) and total remission (upper right). The risk estimate is for the column-defining treatment compared to the row-defining treatment. Statistical significance is defined as 95% confidence intervals that do not overlap 1.0. WM = Western medicine, YXY = Yinxingye injection, SXT = Shuxuetong injection, SK = Shenkang injection, HQ = Huangqi injection, FFDS = Fufangdanshen injection, DZH = Dengzhanhua injection, DH = Danhong injection, DSCX = Danshenchuanxiongqin injection, DS = Danshen injection, and CXQ = Chuanxiongqin injection.

**Table 2:** Network meta-analysis of serum albumin and pairwise meta-analysis of urinary protein excretion.

											Urinary protein excretion (g/24h)
XST + WM	—	—	—	—	—	−0.20 (−0.76, 0.36)	—	—	−**0.60 (**−**1.13,** −**0.07)**	—	−0.76 (−1.69, 0.17)
−0.44 (−3.88, 3.00)	YXY + WM	—	—	—	−0.02 (−0.27, 0.23)	—	—	—	—	—	**−0.77 (−1.33, −0.21)**
−1.81 (−5.27, 1.65)	−1.37 (−4.39, 1.65)	SXT + WM	—	—	**−1.28 (−2.01, −0.55)**	—	—	—	**−1.93 (−2.30, −1.56)**	—	**−1.92 (−2.37, −1.47)**
−1.74 (−4.74, 1.25)	−1.31 (−4.11, 1.50)	0.07 (−2.79, 2.93)	SK + WM	—	—	—	—	—	**−1.70 (−2.58, −0.82)**	—	**−1.36 (−2.38, −0.33)**
−0.51 (−3.23, 2.22)	−0.07 (−2.52, 2.39)	1.30 (−1.23, 3.84)	1.24 (−0.64, 3.12)	HQ + WM	—	—	—	—	−0.53 (−1.09, 0.03)	—	**−0.70 (−0.97, −0.44)**
−0.06 (−3.34, 3.21)	0.38 (−2.26, 3.01)	1.75 (−0.27, 3.76)	1.68 (−0.94, 4.30)	0.44 (−1.81, 2.69)	FFDS + WM	—	**1.06 (0.43, 1.70)**	—	—	—	**−0.99 (−1.66, −0.32)**
0.33 (−2.37, 3.04)	0.77 (−2.32, 3.86)	2.14 (−0.98, 5.27)	2.08 (−0.53, 4.68)	0.84 (−1.44, 3.11)	0.39 (−2.52, 3.31)	DZH + WM	—	—	−0.53 (−1.01, 0.03)	—	−0.26 (−0.54, 0.02)
−1.65 (−4.98, 1.67)	−1.22 (−4.21, 1.78)	0.16 (−2.76, 3.08)	0.09 (−2.58, 2.76)	−1.15 (−3.45, 1.15)	−1.59 (−4.13, 0.95)	−1.99 (−4.95, 0.98)	DH + WM	—	—	—	**−1.52 (−1.88, −1.16)**
0.58 (−2.61, 3.78)	1.02 (−1.92, 3.97)	2.39 (−0.62, 5.41)	2.33 (−0.16, 4.82)	1.09 (−1.01, 3.19)	0.65 (−2.13, 3.43)	0.25 (−2.56, 3.07)	2.24 (−0.58, 5.06)	DSCX + WM	—	**−0.53 (−0.77, −0.30)**	**−1.16 (−1.65, −0.68)**
1.13 (−1.36, 3.62)	1.57 (−1.03, 4.17)	**2.94 (0.34, 5.53)**	**2.87 (0.96, 4.79)**	**1.63 (0.12, 3.14)**	1.19 (−1.18, 3.56)	0.80 (−1.32, 2.91)	**2.78 (0.33, 5.24)**	0.54 (−1.73, 2.82)	DS + WM	—	**−1.16 (−1.66, −0.66)**
1.16 (−1.94, 4.27)	1.60 (−1.25, 4.45)	**2.97 (0.05, 5.90)**	**2.91 (0.53, 5.28)**	1.67 (−0.29, 3.63)	1.23 (−1.45, 3.90)	0.83 (−1.88, 3.55)	**2.82 (0.10, 5.53)**	0.58 (−1.66, 2.81)	0.03 (−2.11, 2.18)	CXQ + WM	**−1.26 (−1.87, −0.66)**
**4.17 (1.58, 6.76)**	**4.61 (2.33, 6.88)**	**5.98 (3.61, 8.34)**	**5.91 (4.27, 7.55)**	**4.67 (3.73, 5.61)**	**4.23 (2.18, 6.28)**	**3.84 (1.73, 5.94)**	**5.82 (3.72, 7.93)**	**3.58 (1.71, 5.46)**	**3.04 (1.75, 4.33)**	**3.00 (1.29, 4.72)**	WM

Serum albumin (g/L)											

The results of network meta-analysis for serum albumin (bottom left) and pairwise meta-analysis for urinary protein excretion (upper right). Treatment estimates are shown as mean differences (95% confidence intervals). The risk estimate is for the column-defining treatment compared to the row-defining treatment. Statistical significance is defined as 95% confidence intervals that do not overlap zero. WM = Western medicine, XST = Xueshuantong injection, YXY = Yinxingye injection, SXT = Shuxuetong injection, SK = Shenkang injection, HQ = Huangqi injection, FFDS = Fufangdanshen injection, DZH = Dengzhanhua injection, DH = Danhong injection, DSCX = Danshenchuanxiongqin injection, DS = Danshen injection, and CXQ = Chuanxiongqin injection.

**Table 3:** Summary of adverse reactions due to the evaluated Chinese herbal injections.

	Experimental group	Control group
CXQ + WM vs. WM	5/89 cases: dizziness, 1 case; hypersomnia, 1 case; dry mouth, 3 cases	0/81 cases

DS + WM vs. WM	1/90 cases: skin rash, 1 case	0/90 cases

DSCX + WM vs. WM	3/123 cases: gastrointestinal adverse reactions, 3 cases	4/123 cases: gastrointestinal adverse reactions, 2 cases; liver dysfunction, 2 cases

DH + WM vs. FFDS + WM	0/60 cases	0/60 cases

DZH + WM vs. WM	0/60 cases	1/60 cases: bleeding, 1 case

HQ + WM vs. WM	18/84 cases Acne, 3 cases; hirsutism, 2 cases; alopecia, 1 case; insomnia, 5 cases; infections, 6 cases; vomiting, 1 case	19/84 cases: acne, 4 cases; hirsutism, 3 cases; alopecia, 4 cases; insomnia, 4 cases; infections, 3 cases; gastrointestinal adverse reaction, 1 case

SK + WM vs. DS + WM	0/50 cases	0/50 cases

SK + WM vs. WM	2/61 cases: headache, 2 cases	0/62 cases

SXT + WM vs. FFDS + WM	0/60 cases	0/60 cases

SXT + WM vs. WM	2/55 cases: subcutaneous haemorrhage, 2 cases	0/55 cases

YXY + WM vs. WM	4/68 cases: gastrointestinal adverse reactions, 2 cases; elevated blood pressure, 2 cases	10/68 cases: gastrointestinal adverse reactions, 7 cases; elevated blood pressure, 3 cases

YXY + WM vs. FFDS + WM	0/30 cases	0/30 cases

XST + WM vs. DZH + WM	7/28 cases: subcutaneous haemorrhage, 6 cases; non-specific adverse reaction, 1 case	4/28 cases: subcutaneous haemorrhage, 4 cases

WM = Western medicine, CXQ = Chuanxiongqin injection, DS = Danshen injection, DSCX = Danshenchuanxiongqin injection, DH = Danhong injection, DZH = Dengzhanhua injection, FFDS = Fufangdanshen injection, HQ = Huangqi injection, SK = Shenkang injection, SXT = Shuxuetong injection, YXY = Yinxingye injection, and XST = Xueshuantong injection.

**Table 4:** SUCRA of Chinese herbal injections for primary nephrotic syndrome.

	Complete remission	Total remission	Serum albumin	Triglycerides	Serum creatinine	Adverse reactions
WM	0.20 (43)	1.70 (43)	0.00 (49)	9.00 (36)	15.0 (23)	53.3 (18)
CXQ + WM	39.0 (4)	37.8 (4)	25.4 (6)	77.8 (5)	51.5 (6)	20.3 (3)
DS + WM	43.2 (9)	46.4 (9)	23.9 (11)	43.4 (9)	80.6 (5)	33.9 (3)
DSCX + WM	36.2 (3)	36.5 (3)	36.5 (4)	22.0 (2)	43.7 (2)	61.7 (2)
DH + WM	82.9 (6)	68.6 (6)	81.9 (4)	43.5 (2)	38.4 (2)	53.1 (1)
DZH + WM	56.6 (4)	82.1 (4)	41.3 (4)	54.2 (5)	73.8 (4)	71.3 (3)
FFDS + WM	24.6 (6)	22.2 (6)	50.2 (7)	63.2 (4)	31.3 (6)	54.9 (3)
HQ + WM	46.7 (15)	54.0 (15)	60.9 (21)	36.9 (12)	80.5 (3)	56.8 (3)
SK + WM	64.9 (6)	70.1 (6)	84.8 (6)	67.1 (2)	53.8 (3)	28.9 (3)
SXT + WM	84.1 (3)	83.0 (3)	85.5 (5)	79.1 (5)	41.6 (2)	33.6 (3)
YXY + WM	71.7 (1)	46.5 (1)	59.7 (3)	27.4 (2)	23.8 (3)	81.1 (3)
XST + WM	—	—	49.9 (3)	76.3 (1)	66.0 (1)	51.1 (1)

Data are shown as SUCRA (number of studies). SUCRA = surface under the cumulative ranking curve, WM = Western medicine, CXQ = Chuanxiongqin injection, DS = Danshen injection, DSCX = Danshenchuanxiongqin injection, DH = Danhong injection, DZH = Dengzhanhua injection, FFDS = Fufangdanshen injection, HQ = Huangqi injection, SK = Shenkang injection, SXT = Shuxuetong injection, YXY = Yinxingye injection, and XST = Xueshuantong injection.

## Data Availability

The data used to support the findings of this study are included within the supplementary materials file.

## Supplementary Materials

Supplementary Materials Table S1: PRISMA checklist for network meta-analysis. Table S2: search strategy. Table S3: characteristics of included studies. Table S4: pairwise meta-analysis of total cholesterol and network meta-analysis of triglyceride. Table S5: pairwise meta-analysis and network meta-analysis of adverse reaction. Table S6: network meta-analysis of serum creatinine. Table S7: inconsistency and heterogeneity test of network meta-analyses.
