# Supplementary material for: Chinese Herbal Injections for Primary Nephrotic Syndrome in Adults: A Systematic Review and Network Meta-Analysis
Source: Evid Based Complement Alternat Med. 2020 Feb 24;2020:1047489. doi: 10.1155/2020/1047489 (PMC7060412; doi:10.1155/2020/1047489)
Supplement: Supplementary Materials — Table S1: PRISMA checklist for network meta-analysis. Table S2: search strategy. Table S3: characteristics of included studies. Table S4: pairwise meta-analysis of total cholesterol and network meta-analysis of triglyceride. Table S5: pairwise meta-analysis and network meta-analysis of adverse reaction. Table S6: network meta-analysis of serum creatinine. Table S7: inconsistency and heterogeneity test of network meta-analyses. [file 1047489.f1.docx]

**Supplementary Table S1-7**

**Table S1. PRISMA checklist for network meta-analysis**

| **Section/Topic** | **Item #** | **Checklist Item** | **Reported on Page #** |
| --- | --- | --- | --- |
| **TITLE** |  |  |  |
| Title | 1 | Identify the report as a systematic review *incorporating a network meta-analysis (or related form of meta-analysis).* | 1 |
|  |  |  |  |
| **ABSTRACT** |  |  |  |
| Structured summary | 2 | Provide a structured summary including, as applicable:  **Background:** main objectives  **Methods:** data sources; study eligibility criteria, participants, and interventions; study appraisal; and *synthesis methods, such as network meta-analysis.*  **Results:** number of studies and participants identified; summary estimates with corresponding confidence/credible intervals; *treatment rankings may also be discussed. Authors may choose to summarize pairwise comparisons against a chosen treatment included in their analyses for brevity.*  **Discussion/Conclusions:** limitations; conclusions and implications of findings.  **Other:** primary source of funding; systematic review registration number with registry name. | 1 |
|  |  |  |  |
| **INTRODUCTION** |  |  |  |
| Rationale | 3 | Describe the rationale for the review in the context of what is already known*, including mention of why a network meta-analysis has been conducted.* | 2 |
| Objectives | 4 | Provide an explicit statement of questions being addressed, with reference to participants, interventions, comparisons, outcomes, and study design (PICOS). | 2 |
|  |  |  |  |
| **METHODS** |  |  |  |
| Protocol and registration | 5 | Indicate whether a review protocol exists and if and where it can be accessed (e.g., Web address); and, if available, provide registration information, including registration number. | 2 |
| Eligibility criteria | 6 | Specify study characteristics (e.g., PICOS, length of follow-up) and report characteristics (e.g., years considered, language, publication status) used as criteria for eligibility, giving rationale. *Clearly describe eligible treatments included in the treatment network, and note whether any have been clustered or merged into the same node (with justification).* | 2-3 |
| Information sources | 7 | Describe all information sources (e.g., databases with dates of coverage, contact with study authors to identify additional studies) in the search and date last searched. | 2 |
| Search | 8 | Present full electronic search strategy for at least one database, including any limits used, such that it could be repeated. | 2 |
| Study selection | 9 | State the process for selecting studies (i.e., screening, eligibility, included in systematic review, and, if applicable, included in the meta-analysis). | 2 |
| Data collection process | 10 | Describe method of data extraction from reports (e.g., piloted forms, independently, in duplicate) and any processes for obtaining and confirming data from investigators. | 3 |
| Data items | 11 | List and define all variables for which data were sought (e.g., PICOS, funding sources) and any assumptions and simplifications made. | 2-3 |
| **Geometry of the network** | **S1** | Describe methods used to explore the geometry of the treatment network under study and potential biases related to it. This should include how the evidence base has been graphically summarized for presentation, and what characteristics were compiled and used to describe the evidence base to readers. | 3 |
| Risk of bias within individual studies | 12 | Describe methods used for assessing risk of bias of individual studies (including specification of whether this was done at the study or outcome level), and how this information is to be used in any data synthesis. | 3 |
| Summary measures | 13 | State the principal summary measures (e.g., risk ratio, difference in means). *Also describe the use of additional summary measures assessed, such as treatment rankings and surface under the cumulative ranking curve (SUCRA) values, as well as modified approaches used to present summary findings from meta-analyses.* | 3 |
| Planned methods of analysis | 14 | Describe the methods of handling data and combining results of studies for each network meta-analysis. This should include, but not be limited to:   - *Handling of multi-arm trials;* - *Selection of variance structure;* - *Selection of prior distributions in Bayesian analyses; and* - *Assessment of model fit.* | 3 |
| **Assessment of Inconsistency** | **S2** | Describe the statistical methods used to evaluate the agreement of direct and indirect evidence in the treatment network(s) studied. Describe efforts taken to address its presence when found. | 3 |
| Risk of bias across studies | 15 | Specify any assessment of risk of bias that may affect the cumulative evidence (e.g., publication bias, selective reporting within studies). | 3 |
| Additional analyses | 16 | Describe methods of additional analyses if done, indicating which were pre-specified. This may include, but not be limited to, the following:   - Sensitivity or subgroup analyses; - Meta-regression analyses; - *Alternative formulations of the treatment network; and* - *Use of alternative prior distributions for Bayesian analyses (if applicable).* | 3 |
|  |  |  |  |
| **RESULTS†** |  |  |  |
| Study selection | 17 | Give numbers of studies screened, assessed for eligibility, and included in the review, with reasons for exclusions at each stage, ideally with a flow diagram. | 3-4 |
| **Presentation of network structure** | **S3** | Provide a network graph of the included studies to enable visualization of the geometry of the treatment network. | 5 |
| **Summary of network geometry** | **S4** | Provide a brief overview of characteristics of the treatment network. This may include commentary on the abundance of trials and randomized patients for the different interventions and pairwise comparisons in the network, gaps of evidence in the treatment network, and potential biases reflected by the network structure. | 4-5 |
| Study characteristics | 18 | For each study, present characteristics for which data were extracted (e.g., study size, PICOS, follow-up period) and provide the citations. | 3-4 |
| Risk of bias within studies | 19 | Present data on risk of bias of each study and, if available, any outcome level assessment. | 5 |
| Results of individual studies | 20 | For all outcomes considered (benefits or harms), present, for each study: 1) simple summary data for each intervention group, and 2) effect estimates and confidence intervals. *Modified approaches may be needed to deal with information from larger networks.* | 6-15 |
| Synthesis of results | 21 | Present results of each meta-analysis done, including confidence/credible intervals. *In larger networks, authors may focus on comparisons versus a particular comparator (e.g. placebo or standard care), with full findings presented in an appendix. League tables and forest plots may be considered to summarize pairwise comparisons.* If additional summary measures were explored (such as treatment rankings), these should also be presented. | 6-15 |
| **Exploration for inconsistency** | **S5** | Describe results from investigations of inconsistency. This may include such information as measures of model fit to compare consistency and inconsistency models, *P* values from statistical tests, or summary of inconsistency estimates from different parts of the treatment network. | 14 |
| Risk of bias across studies | 22 | Present results of any assessment of risk of bias across studies for the evidence base being studied. | 14-15 |
| Results of additional analyses | 23 | Give results of additional analyses, if done (e.g., sensitivity or subgroup analyses, meta-regression analyses*, alternative network geometries studied, alternative choice of prior distributions for Bayesian analyses,* and so forth). | 14-15 |
|  |  |  |  |
| **DISCUSSION** |  |  |  |
| Summary of evidence | 24 | Summarize the main findings, including the strength of evidence for each main outcome; consider their relevance to key groups (e.g., healthcare providers, users, and policy-makers). | 15 |
| Limitations | 25 | Discuss limitations at study and outcome level (e.g., risk of bias), and at review level (e.g., incomplete retrieval of identified research, reporting bias). *Comment on the validity of the assumptions, such as transitivity and consistency. Comment on any concerns regarding network geometry (e.g., avoidance of certain comparisons).* | 16 |
| Conclusions | 26 | Provide a general interpretation of the results in the context of other evidence, and implications for future research. | 17 |
|  |  |  |  |
| **FUNDING** |  |  |  |
| Funding | 27 | Describe sources of funding for the systematic review and other support (e.g., supply of data); role of funders for the systematic review. This should also include information regarding whether funding has been received from manufacturers of treatments in the network and/or whether some of the authors are content experts with professional conflicts of interest that could affect use of treatments in the network. | 17 |

PICOS = population, intervention, comparators, outcomes, study design.

**Table S2. Search strategy**

**1. Databases**

| English databases | Chinese databases |
| --- | --- |
| PubMed | China National Knowledge Infrastructure database (CNKI) |
| Embase | WangFang database |
| Cochrane Central Register of Controlled Trials (CENTRAL) | Chinese Scientific and Technology Journal Database (VIP) |
|  | China Biology Medicine database (CBM) |

**2. Search strategy of** **Pubmed**

#1 nephrotic syndrome [MeSH Terms]

#2 nephrotic syndrome [All Fields]

#3 nephro* [Title/Abstract]

#4 minimal change disease [Title/Abstract]

#5 minimal change nephropathy [Title/Abstract]

#6 membranous nephropathy [Title/Abstract]

#7 IgA nephropathy [Title/Abstract]

#8 focal segmental glomerulosclerosis [Title/Abstract]

#9 mesangial proliferative glomerulonephritis [Title/Abstract]

#10 membranoproliferative glomerulonephritis [Title/Abstract]

#11 #1 OR #2 OR #3 OR #4 OR #5 OR #6 OR #7 OR #8 OR #9 OR #10

#12 Traditional Chinese medicine [All Fields]

#13 Traditional Chinese medicine [MeSH Terms]

#14 Chinese herbal injection [All Fields]

#15 huangqi [Title/Abstract]

#16 astragalus [Title/Abstract]

#17 honghua [Title/Abstract]

#18 safflower [Title/Abstract]

#19 danshen [Title/Abstract]

#20 salvia miltiorrhiza [Title/Abstract]

#21 xuesaitong [Title/Abstract]

#22 shenkang [Title/Abstract]

#23 shuxuetong [Title/Abstract]

#24 xueshuantong [Title/Abstract]

#25 panax notoginseng saponins [Title/Abstract]

#26 shuizhi dilong [Title/Abstract]

#27 shuizhidilong [Title/Abstract]

#28 earthworm leech [Title/Abstract]

#29 dengzhanxixin [Title/Abstract]

#30 scutellarin caffeate [Title/Abstract]

#31 xiangdan [Title/Abstract]

#32 danshen jiangxiang [Title/Abstract]

#33 salvia dalbergia [Title/Abstract]

#34 danhong [Title/Abstract]

#35 danshenchuanxiongqin [Title/Abstract]

#36 danshen – chuanxiongqin [Title/Abstract]

#37 chuanxiongqin [Title/Abstract]

#38 ligustrazine [Title/Abstract]

#39 salvia safflower [Title/Abstract]

#40 shengmai [Title/Abstract]

#41 shenmai [Title/Abstract]

#42 shenfu [Title/Abstract]

#43 shuxuening [Title/Abstract]

#44 ginkgolide [Title/Abstract]

#45 yinxingye [Title/Abstract]

#46 #12 OR #13 OR #14 OR #15 OR #16 OR #17 OR #18 OR #19 OR #20 OR #21 OR #22 OR #23 OR #24 OR #25 OR #26 OR #27 OR #28 OR #29 OR #30 OR #31 OR #32 OR #33 OR #34 OR #35 OR #36 OR #37 OR #38 OR #39 OR #40 OR #41 OR #42 OR #43 OR #44 OR #45

#47 random* [All Fields]

#48 randomized controlled trial [Publication Type]

#49 controlled clinical trial [Publication Type]

#50 trial [Title/Abstract]

#51 #47 OR #48 OR #49 OR #50

#52 #11 AND #46 AND #51

**3. Search strategy of Embase**

#1 'nephrotic syndrome'/exp

#2 nephrotic syndrome

#3 Minimal change disease':ab,ti

#4 'Membranous nephropathy':ab,ti

#5 'IgA nephropathy':ab,ti

#6 'Focal segmental glomerulosclerosis':ab,ti

#7 'Mesangial proliferative glomerulonephritis':ab,ti

#8 'Membranoproliferative glomerulonephritis':ab,ti

#9 'Minimal change nephropathy':ab,ti

#10 #1 or #2 or #3 or #4 or #5 or #6 or #7 or #8 or #9

#11 Traditional Chinese medicine

#12 Chinese herbal injection

#13 astragalus:ab,ti

#14 honghua:ab,ti

#15 danshen:ab,ti

#16 'salvia miltiorrhiza':ab,ti

#17 huangqi:ab,ti

#18 safflower:ab,ti

#19 xuesaitong:ab,ti

#20 shenkang:ab,ti

#21 shuxuetong:ab,ti

#22 xueshuantong:ab,ti

#23 'panax notoginseng saponins':ab,ti

#24 'shuizhi dilong':ab,ti

#25 shuizhidilong:ab,ti

#26 earthworm:ab,ti AND leech:ab,ti

#27 dengzhanxixin:ab,ti

#28 scutellarin:ab,ti AND caffeate:ab,ti

#29 xiangdan:ab,ti

#30 danshen:ab,ti AND jiangxiang:ab,ti

#31 salvia:ab,ti AND dalbergia:ab,ti

#32 danhong:ab,ti

#33 danshenchuanxiongqin:ab,ti

#34 danshen chuanxiongqin':ab,ti

#35 chuanxiongqin:ab,ti

#36 ligustrazine:ab,ti

#37 salvia:ab,ti AND safflower:ab,ti

#38 shengmai:ab,ti

#39 shenfu:ab,ti

#40 shenmai:ab,ti

#41 shuxuening:ab,ti

#42 ginkgolide:ab,ti

#43 yinxingye:ab,ti

#44 #11 OR #12 OR #13 OR #14 OR #15 OR #16 OR #17 OR #18 OR #19 OR #20 OR #21 OR #22 OR #23 OR #24 OR #25 OR #26 OR #27 OR #28 OR #29 OR #30 OR #31 OR #32 OR #33 OR #34 OR #35 OR #36 OR #37 OR #38 OR #39 OR #40 OR #41 OR #42 OR #43

#45 'randomized controlled trial'/exp

#46 random*

#47 trial:ab,ti

#48 #45 OR #46 OR #47

#49 #10 AND #44 AND #48

**4. Search strategy of CENTRAL**

#1 MeSH descriptor: [Nephrotic Syndrome] explode all trees

#2 "nephrotic syndrome" (Word variations have been searched)

#3 (Minimal change disease) OR (Membranous nephropathy) OR (IgA Nephropathy) OR (Focal Segmental Glomerulosclerosis) OR (Mesangial proliferative glomerulonephritis) OR (Membranoproliferative Glomerulonephritis) (Word variations have been searched)

#4 #1 or #2 or #3

#5 (Traditional Chinese Medicine) OR (Chinese herbal injection) (Word variations have been searched)

#6 (astragalus):ti,ab,kw OR (huangqi):ti,ab,kw OR (Salvia miltiorrhiza):ti,ab,kw OR (danshen):ti,ab,kw OR (honghua):ti,ab,kw (Word variations have been searched)

#7 (safflower):ti,ab,kw OR (shenkang):ti,ab,kw OR (shuxuetong):ti,ab,kw OR (xuesaitong):ti,ab,kw in Trials (Word variations have been searched)

#8 (xueshuantong):ti,ab,kw OR (panax notoginseng saponins):ti,ab,kw OR (shuizhi-dilong):ti,ab,kw OR (shuizhidilong):ti,ab,kw OR (Earthworm and Leech):ti,ab,kw (Word variations have been searched)

#9 (dengzhanxixin):ti,ab,kw OR (Scutellarin and caffeate):ti,ab,kw OR (xiangdan):ti,ab,kw OR (danshen and jiangxiang):ti,ab,kw OR (Salvia and Dalbergia):ti,ab,kw (Word variations have been searched)

#10 (danhong):ti,ab,kw OR (danshenchuanxiongqin):ti,ab,kw OR (danshen-chuanxiongqin):ti,ab,kw OR (ligustrazine):ti,ab,kw OR (Salvia and safflower):ti,ab,kw (Word variations have been searched)

#11 (shengmai):ti,ab,kw OR (shenmai):ti,ab,kw OR (shenfu):ti,ab,kw OR (shuxuening):ti,ab,kw OR (ginkgolide):ti,ab,kw (Word variations have been searched)

#12 #5 or #6 or #7 or #8 or #9 or #10 or 11

#13 #4 and #12

**4. Search strategy of** **Chinese databases**

The similar retrieval strategies were performed in four Chinese databases.

**Table S3. Characteristics of included studies**

| Study | Sample | | Gender | | Intervention (T/C) | Course  (day) | Selection bias | | Blinding | Attrition bias | Reporting  bias | Other bias | outcomes |
| --- | --- | --- | --- | --- | --- | --- | --- | --- | --- | --- | --- | --- | --- |
|  | T | C | M | F |  |  | Random | Allocation concealment |  |  |  |  |  |
| Bai YW 2004 | 31 | 32 | 37 | 26 | HQ+WM/WM | 28 | Unclear | High | Unclear | Low | Unclear | Unclear | (1)(2)(3)(4)  (7)(8) |
| Bian BJ 2016 | 35 | 36 | 38 | 33 | SK+WM/WM | 21 | Unclear | High | Unclear | Low | High | Unclear | (1)(2)(3)(4)  (5)(6)(7)(8) |
| Bu LY 2014 | 42 | 42 | 47 | 37 | SXT+WM/WM | 14 | Unclear | High | Unclear | Low | Unclear | Unclear | (1)(2)(3)(4)  (5)(6)(7)(8) |
| Cao XW 2009 | 36 | 32 | 38 | 30 | DH+WM/WM | 28 | Unclear | High | Unclear | Low | Unclear | Unclear | (1)(2)(3)(4)  (6)(7)(8) |
| Chen LP 2004 | 30 | 30 | 42 | 18 | HQ+WM/WM | 30 | Unclear | High | Unclear | Low | Unclear | Unclear | (3) |
| Dai H 2009 | 33 | 33 | 45 | 21 | CXQ+WM/WM | 21 | Unclear | High | Unclear | Low | Unclear | Unclear | (3)(4)  (6)(7)(8) |
| Deng YH 2010 | 26 | 25 | 35 | 16 | CXQ+WM/WM | 28 | Unclear | High | Unclear | Low | Unclear | Unclear | (1)(2)(3)(4)  (7)(8) |
| Fan RF 2001 | 36 | 20 | 34 | 22 | HQ+WM/WM | 28 | Unclear | High | Unclear | Low | Unclear | Unclear | (3)(4)  (7)(8) |
| Fen X 2002 | 30 | 26 | 30 | 26 | DS+WM/WM | 28 | Unclear | High | Unclear | Low | Unclear | Unclear | (1)(2)(3)(4)  (6)(7)(8) |
| Fen Y 2010 | 60 | 60 | 61 | 59 | DH+WM/FFDS+WM | 28 | Low | High | Unclear | Low | Unclear | Unclear | (1)(2)(3)(4)  (5)(6)(8) |
| Feng JC 2012 | 20 | 18 | 25 | 13 | DH+WM/WM | 42 | Unclear | High | Unclear | Low | Unclear | Unclear | (1)(2)(3)(4)  (8) |
| Fu P 2003 | 30 | 30 | 28 | 32 | DZH+WM/WM | 14 | Low | High | Unclear | Low | Unclear | Unclear | (3)(4)  (5)(7)(8) |
| Fu Q 2019 | 60 | 60 | 65 | 55 | DS+WM/WM | 14 | Unclear | High | Unclear | Low | Unclear | Unclear | (1)(2)(3)  (5)(6) |
| Gao YX 2004 | 30 | 20 | 33 | 17 | DZH+WM/WM | 30 | Unclear | High | Unclear | Low | Unclear | Unclear | (1)(2)(3)  (6)(7)(8) |
| Gong X 2014 | 32 | 32 | 29 | 35 | HQ+WM/WM | 28 | Unclear | High | Unclear | Low | Unclear | Unclear | (3)(4)  (7)(8) |
| Hu PY 2014 | 29 | 27 | 37 | 19 | CXQ+WM/WM | 45 | Unclear | High | Unclear | Low | Unclear | Unclear | (1)(2)(3)(4)  (6)(8) |
| Hu Y 2018 | 29 | 30 | 32 | 27 | HQ+WM/WM | 42 | Unclear | High | Unclear | Low | Unclear | Unclear | (2) |
| Jia SL 2017 | 38 | 38 | 43 | 33 | SK+WM/WM | 14 | Unclear | High | Unclear | Low | Unclear | Unclear | (3)(4) |
| Jiang Y 2008 | 23 | 20 | 18 | 25 | HQ+WM/WM | 28 | Unclear | High | Unclear | Low | High | Unclear | (1)(2)(3)(4)  (7)(8) |
| Ke H 2011 | 42 | 39 | 44 | 37 | HQ+WM/WM | 56 | Unclear | High | Unclear | Low | Unclear | Unclear | (1)(2)(3)(4) |
| Lei P 2018 | 41 | 41 | 53 | 29 | DZH+WM/WM | 14 | Low | High | Unclear | Low | Unclear | Unclear | (3) |
| Li T 2004 | 71 | 60 | 77 | 54 | HQ+WM/WM | 28 | Unclear | High | Unclear | Low | Unclear | Unclear | (3)(4)(7)(8) |
| Li XN 2017 | 42 | 42 | 38 | 46 | FFDS+WM/WM | 10 | Unclear | High | Unclear | Low | Unclear | Unclear | (1)(2)(3)(4) |
| Li XY 2007 | 56 | 60 | 62 | 54 | SXT+WM/DS+WM | 60 | Unclear | High | Unclear | Low | Unclear | Unclear | (3)(7)(8) |
| Li Z1999 | 14 | 14 | 17 | 11 | HQ+WM/WM | 30 | Unclear | High | Unclear | Low | Unclear | Unclear | (3)(4)(8) |
| Li ZF 2001 | 20 | 19 | 18 | 21 | CXQ+WM/WM | 21 | Unclear | High | Unclear | Low | Unclear | Unclear | (3)(4)(5)(8) |
| Liao YJ 2009 | 40 | 40 | 41 | 39 | HQ+WM/WM | 7 | Unclear | High | Unclear | Low | Unclear | Unclear | (1)(2) |
| Liu LS 2003 | 25 | 25 | 29 | 21 | HQ+WM/WM | 56 | Low | High | Unclear | Low | Unclear | Unclear | (3)(4)(7)(8) |
| Liu ML 2001 | 14 | 12 | 16 | 10 | HQ+WM/WM | 15 | Unclear | High | Unclear | Low | Unclear | Unclear | (1)(2)(3)(4)  (6)(7)(8) |
| Liu S 2014 | 20 | 20 | 24 | 16 | HQ+WM/WM | 28 | Unclear | High | Unclear | Low | Unclear | Unclear | (1)(2)(3)(4) |
| Liu WL 2008 | 26 | 22 | 23 | 25 | HQ+WM/DS+WM | 28 | Low | High | Unclear | Low | Unclear | Unclear | (1)(2)(3)(4)  (7)(8) |
| Liu YH 2016 | 46 | 46 | 54 | 38 | DH+WM/WM | 14 | Unclear | High | Unclear | Low | Unclear | Unclear | (1)(2) |
| Liu YL 2007 | 24 | 16 | 25 | 15 | HQ+WM/WM | 30 | Unclear | High | Unclear | Low | Unclear | Unclear | (1)(2)(3)(4) |
| Liu YP 2008 | 40 | 40 | 48 | 32 | HQ+WM/WM | 45 | Unclear | High | Unclear | Low | Unclear | Unclear | (1)(2)(3)(4)  (5) |
| Lu J 2006 | 26 | 26 | 24 | 28 | YXY+WM/WM | 28 | Unclear | High | Unclear | Low | Unclear | Unclear | (3)(4)  (5)(6)(7)(8) |
| Ma TA 2002 | 26 | 21 | 27 | 20 | DZH+WM/WM | 28 | Unclear | High | Unclear | Low | Unclear | Unclear | (1)(2)(3)  (6)(7)(8) |
| Mo ZY 2004 | 26 | 21 | 31 | 16 | HQ+WM/WM | 20 | Unclear | High | Unclear | Low | Unclear | Unclear | (3)(4)  (7)(8) |
| Nie XD 2009 | 28 | 28 | 24 | 32 | XST+WM/DZH+WM | 28 | Unclear | High | Unclear | Low | Unclear | Unclear | (3)(4)  (5)(6)(7)(8) |
| Niu K 2011 | 23 | 22 | 22 | 23 | SK+WM/WM | 14 | Unclear | High | Unclear | Low | Unclear | Unclear | (3)(4)(5) |
| Niu XC 2004 | 30 | 26 | 32 | 24 | SXT+WM/FFDS+WM | 14 | Unclear | High | Unclear | Low | Unclear | Unclear | (3)(4)(7)(8) |
| Pan HM 2013 | 48 | 50 | 45 | 53 | FFDS+WM/WM | 28 | Unclear | High | Unclear | Low | Unclear | Unclear | (1)(2) |
| Pan LP 2009 | 30 | 30 | 31 | 29 | DSCX+WM/CXQ+WM | 14 | Unclear | High | Unclear | Low | Unclear | Unclear | (1)(2)(3)(4)  (6) |
| Shen Y 2002 | 28 | 28 | 32 | 24 | HQ+WM/WM | 70 | Unclear | High | Unclear | Low | High | Unclear | (3)(4)(7) |
| Shen Y 2010 | 24 | 24 | 32 | 16 | DSCX+WM/WM | 56 | Unclear | High | Unclear | Low | High | Unclear | (3)(4)(7)(8) |
| Song LQ 2010 | 28 | 30 | 31 | 27 | SXT+WM/DS+WM | 60 | Low | High | Unclear | Low | Unclear | Unclear | (1)(2)(3)(4)  (7)(8) |
| Sun CF 2015 | 48 | 48 | 57 | 39 | HQ+WM/WM | 10 | Unclear | High | Unclear | Low | Unclear | Unclear | (1)(2)(3)(4)  (7)(8) |
| Wan GX 2006 | 30 | 20 | 33 | 17 | CXQ+WM/WM | 30 | Unclear | High | Unclear | Low | Unclear | Unclear | (1)(2)(3)  (6)(7)(8) |
| Wang H 2006 | 36 | 32 | 38 | 30 | DS+WM/WM | 28 | Unclear | High | Unclear | Low | Unclear | Unclear | (1)(2)(3)(4)  (6)(8) |
| Wang M 2015 | 40 | 40 | 56 | 24 | SK+WM/WM | 14 | Low | High | Unclear | Low | Unclear | Unclear | (1)(2)(3)(4)  (7)(8) |
| Wang WF 2007 | 30 | 30 | 36 | 24 | DS+WM/WM | 28 | Low | High | Unclear | Low | Unclear | Unclear | (3)(5)(6)(7) |
| Wang XH 2010 | 15 | 15 | 20 | 10 | SXT+WM/WM | 14 | Low | High | Unclear | Low | Unclear | Unclear | (3)(5) |
| Wang Y 2007 | 30 | 30 | 31 | 29 | YXY+WM/FFDS+WM | 14 | Unclear | High | Unclear | Low | Unclear | Unclear | (3)(4)(5)(6) |
| Wang YF 2002 | 30 | 28 | 33 | 25 | HQ+WM/WM | 28 | Unclear | High | Unclear | Low | Unclear | Unclear | (1)(2)(3)(4)  (6)(8) |
| Wu CM 2010 | 43 | 40 | 46 | 37 | SXT+WM/WM | 60 | Unclear | High | Unclear | Low | Unclear | Unclear | (1)(2) |
| Wu FL 2003 | 40 | 33 | 41 | 32 | CXQ+WM/WM | 21 | Unclear | High | Unclear | Low | Unclear | Unclear | (5)(6)(7)(8) |
| Wu G 2006 | 29 | 29 | 33 | 25 | CXQ+WM/WM | 14 | Unclear | High | Unclear | Low | Unclear | Unclear | (3)(4)  (5)(6)(7)(8) |
| Wu WB 1999 | 60 | 60 | 68 | 52 | HQ+WM/WM | 15 | Unclear | High | Unclear | Low | Unclear | Unclear | (3) |
| Xie QK 2011 | 50 | 50 | 70 | 30 | SK+WM/DS+WM | 28 | Unclear | High | Unclear | Low | Unclear | Unclear | (1)(2)(3)(4)  (5)(6) |
| Xiong YM 2006 | 40 | 36 | 53 | 23 | DS+WM/WM | 50 | Unclear | High | Unclear | Low | Unclear | Unclear | (1)(2)(3)(4)  (7)(8) |
| Xu L 2009 | 40 | 40 | 56 | 24 | SXT+WM/WM | 21 | Unclear | High | Unclear | Low | Unclear | Unclear | (3)(4)  (5)(7)(8) |
| Xu ML 2018 | 39 | 39 | 51 | 27 | SK+WM/WM | 28 | Unclear | High | Unclear | Low | Unclear | Unclear | (1)(2)(3)(4)  (5)(6) |
| Xuan YH 2005 | 34 | 34 | 34 | 34 | SXT+WM/FFDS+WM | 21 | Unclear | High | Unclear | Low | Unclear | Unclear | (3)(4)  (6)(7)(8) |
| Yan R 2014 | 80 | 80 | 109 | 51 | DSCX+WM/WM | 14 | Low | High | Unclear | Low | Unclear | Unclear | (1)(2)(3)(4)  (5) |
| Yan WW 2010 | 20 | 20 | 20 | 20 | XST+WM/WM | 56 | Unclear | High | Unclear | Low | Unclear | Unclear | (3)(4)(8) |
| Yang AC 2011 | 30 | 26 | 31 | 25 | DH+WM/WM | 56 | Unclear | High | Unclear | Low | Unclear | Unclear | (1)(2)(3)(4)  (7)(8) |
| Yang BK 2010 | 44 | 44 | 50 | 38 | DH+WM/WM | - | Unclear | High | Unclear | Low | High | Unclear | (1)(2) |
| Yang XD 2002 | 19 | 19 | 22 | 16 | HQ+WM/WM | 28 | Unclear | High | Unclear | Low | Unclear | Unclear | (1)(2)(3)(4)  (5) |
| Yao YC 2007 | 44 | 43 | 40 | 47 | DS+WM/WM | 28 | Unclear | High | Unclear | Low | Unclear | Unclear | (3)(4)(7)(8) |
| Ye WB 2006 | 36 | 20 | 34 | 22 | FFDS+WM/WM | 28 | Unclear | High | Unclear | Low | High | Unclear | (1)(2)(3)  (7)(8) |
| Yu P 2005 | 35 | 25 | 36 | 24 | HQ+WM/WM | 14 | Unclear | High | Unclear | Low | Unclear | Unclear | (7)(8) |
| Yu XM 2010 | 38 | 27 | 44 | 21 | DS+WM/WM | 30 | Unclear | High | Unclear | Low | Unclear | Unclear | (1)(2)(3)(4)  (7)(8) |
| Yuan F 2009 | 26 | 22 | 26 | 22 | FFDS+WM/WM | 28 | Unclear | High | Unclear | Low | Unclear | Unclear | (3)(4)  (6)(7)(8) |
| Zhan H 2015 | 60 | 60 | 71 | 49 | HQ+WM/WM | 28 | Unclear | High | Unclear | Low | Unclear | Unclear | (3)(4) |
| Zhan JH 2005 | 16 | 12 | 19 | 9 | DZH+WM/WM | 30 | Unclear | High | Unclear | Low | High | Unclear | (1)(2)(3)(4) |
| Zhan JH 2011 | 30 | 30 | 29 | 31 | XST+WM/DS+WM | 60 | Unclear | High | Unclear | Low | Unclear | Unclear | (3)(4) |
| Zhang B 2011 | 30 | 30 | 28 | 32 | DZH+WM/WM | 14 | Unclear | High | Unclear | Low | Unclear | Unclear | (1)(2)(3)(4)  (5)(6)(7)(8) |
| Zhang L 2016 | 44 | 44 | 47 | 41 | HQ+WM/WM | 28 | Unclear | High | Unclear | Low | Unclear | Unclear | (1)(2) |
| Zhang X 2012 | 19 | 20 | 24 | 15 | SK+WM/WM | 15-30 | Unclear | High | Unclear | Low | High | Unclear | (1)(2) |
| Zhang XW 2010 | 44 | 44 | 47 | 41 | HQ+WM/WM | 10 | Unclear | High | Unclear | Low | Unclear | Unclear | (1)(2)(3)(4)  (6)(8) |
| Zhao J 2007 | 60 | 60 | 62 | 58 | SXT+WM/FFDS+WM | 14 | Unclear | High | Unclear | Low | Unclear | Unclear | (1)(2)(3)(4)  (5)(6)(8) |
| Zhou YH 2018 | 43 | 43 | 59 | 27 | DSCX+WM/WM | 14 | Low | High | Unclear | Low | Unclear | Unclear | (1)(2)(3)(4)  (5)(6)(7)(8) |
| Zhu SP 1998 | 30 | 26 | 37 | 19 | FFDS+WM/WM | 28 | Unclear | High | Unclear | Low | Unclear | Unclear | (1)(2)(3)(8) |
| Zhuang L 2011 | 18 | 21 | 25 | 14 | HQ+WM/WM | 28 | Unclear | High | Unclear | Low | High | Unclear | (3)(4) |
| Zhuang L 2012 | 45 | 45 | 50 | 40 | SK+WM/WM | 42 | Unclear | High | Unclear | Low | Unclear | Unclear | (1)(2)(5) |
| Gong SF | 30 | 28 | 48 | 42 | DS+WM/WM | 28 | Unclear | High | Unclear | Low | Unclear | Unclear | (3)(4)(7)(8) |
|  | 32 |  |  |  | HQ+WM/WM |  |  |  |  |  |  |  |  |

T = Experimental group, C = Control group, M = Male, F = Female. WM = Western medicine, CXQ = Chuanxiongqin injection, DS = Danshen injection, DSCX = Danshenchuanxiongqin injection, DH = Danhong injection, DZH = Dengzhanhua injection, FFDS = Fufangdanshen injection, HQ = Huangqi injection, SK = Shenkang injection, SXT = Shuxuetong injection, YXY = Yinxingye injection, XST = Xueshuantong injection, (1) Complete remission, (2) Total remission, (3) 24-hour urinary protein excretion, (4) Serum albumin, (5) Adverse reaction, (6) Serum creatinine, (7) Total cholesterol, (8) Triglyceride.

**Table S4. Pairwise meta-analysis of total cholesterol and network meta-analysis of triglyceride**

| XST+WM | -1.09  (-2.92, 0.75) | -0.15  (-1.89, 1.59) | -0.34  (-2.18, 1.51) | -0.88  (-2.51, 0.76) | -0.43  (-2.19, 1.32) | -0.60  (-2.02, 0.82) | -0.80  (-2.67, 1.07) | -1.22  (-3.05, 0.61) | -0.76  (-2.42, 0.90) | -0.19  (-1.88, 1.51) | -1.36  (-2.94 , 0.23) |
| --- | --- | --- | --- | --- | --- | --- | --- | --- | --- | --- | --- |
| - | YXY+WM | 0.93  (-0.24, 2.10) | 0.75  (-0.57, 2.06) | 0.21  (-0.80, 1.21) | 0.65  (-0.54, 1.84) | 0.49  (-0.67, 1.64) | 0.29  (-1.07, 1.64) | -0.13  (-1.43, 1.17) | 0.33  (-0.71, 1.37) | 0.90  (-0.20, 2.00) | -0.27  (-1.19, 0.65) |
| - | - | SXT+WM | -0.19  (-1.37, 1.00) | -0.72  (-1.53, 0.08) | -0.28  (-1.04, 0.49) | -0.45  (-1.45, 0.55) | -0.65  (-1.88, 0.59) | -1.06  (-2.23, 0.10) | -0.60  (-1.32, 0.11) | -0.03  (-0.97, 0.91) | **-1.20**  **(-1.92, -0.48)** |
| - | - | - | SK+WM | -0.54  (-1.57, 0.49) | -0.09  (-1.31, 1.12) | -0.26  (-1.44, 0.91) | -0.46  (-1.84, 0.92) | -0.88  (-2.19, 0.44) | -0.42  (-1.49, 0.65) | 0.15  (-0.97, 1.27) | **-1.02**  **(-1.96, -0.07)** |
| - | - | - | - | HQ+WM | 0.45  (-0.40, 1.30) | 0.28  (-0.52, 1.08) | 0.08  (-1.00, 1.16) | -0.34  (-1.34, 0.66) | 0.12  (-0.48, 0.72) | 0.69  (-0.03, 1.42) | **-0.48**  **(-0.88, -0.08)** |
| - | - | **-1.50**  **(-1.90, -1.11)** | - | - | FFDS+WM | -0.17  (-1.20, 0.86) | -0.37  (-1.62, 0.89) | -0.78  (-1.97, 0.40) | -0.33  (-1.16, 0.51) | 0.24  (-0.73, 1.21) | **-0.93**  **(-1.68, -0.17)** |
| 0.19  (-0.61, 0.99) | - | - | - | - |  | DZH+WM | -0.20  (-1.42, 1.02) | -0.62  (-1.77, 0.53) | -0.16  (-1.01, 0.69) | 0.41  (-0.51, 1.33) | **-0.76**  **(-1.45, -0.06)** |
| - | - | - | - | - | **1.32**  **(0.75, 1.89)** | - | DH+WM | -0.42  (-1.77, 0.94) | 0.04  (-1.07, 1.15) | 0.61  (-0.56, 1.78) | -0.56  (-1.56, 0.44) |
| - | - | - | - | - | - | - | - | DSCX+WM | 0.46  (-0.58, 1.50) | 1.03  (-0.07, 2.13) | -0.14  (-1.06, 0.77) |
| - | - | -1.00  (-3.25, 1.25) | - | **-1.70**  **(-3.39, -0.01)** | - | - | - | - | DS+WM | 0.57  (-0.21, 1.35) | **-0.60**  **(-1.09, -0.11)** |
| - | - | - | - |  | - | - | - | - | - | CXQ+WM | **-1.17**  **(-1.77, -0.57)** |
| -0.12  (-0.99, 0.75) | **-2.17**  **(-2.75, -1.58))** | -0.68  (-1.44, 0.08) | -1.67  (-3.49, 0.16) | **-1.27**  **(-1.57, -0.97)** | **-1.60**  **(-1.95, -1.25)** | **-1.42**  **(-2.12, -0.71)** | 0.14  (-1.35, 1.63) | -0.44  (-1.05, 0.16) | **-1.46**  **(-2.22, -0.69)** | **-1.47**  **(-2.28, -0.66)** | WM |

The results of pairwise meta-analysis for total cholesterol (bottom left) and network meta-analysis for triglyceride (upper right). Treatment estimates are shown as mean differences (95% confidence intervals). The risk estimate is for the column-defining treatment compared to the row-defining treatment. Statistical significance is defined as 95% confidence intervals that do not overlap zero. WM = Western medicine, XST = Xueshuantong injection, YXY = Yinxingye injection, SXT = Shuxuetong injection, SK = Shenkang injection, HQ = Huangqi injection, FFDS = Fufangdanshen injection, DZH = Dengzhanhua injection, DH = Danhong injection, DSCX = Danshenchuanxiongqin injection, DS = Danshen injection, CXQ = Chuanxiongqin injection.

**Table S5. Pairwise meta-analysis and network meta-analysis of adverse reaction**

| XST+WM |  |  |  |  |  |  |  |  |  |  | - |
| --- | --- | --- | --- | --- | --- | --- | --- | --- | --- | --- | --- |
| 2.53 (0.12, 55.24) | YXY+WM |  |  |  | NS |  |  |  |  |  | 0.34 (0.09, 1.18) |
| 0.48 (0.01, 18.09) | 0.19 (0.02, 2.18) | SXT+WM |  |  | NS | 2.00 (0.51, 7.80) |  |  |  |  | 5.74 (0.25, 130.37) |
| 0.38 (0.01, 13.45) | 0.15 (0.01, 1.73) | 0.79 (0.03, 17.77) | SK+WM |  |  |  |  |  | NS |  | 5.73 (0.26, 126.42) |
| 1.11 (0.06, 21.61) | 0.44 (0.10, 1.84) | 2.32 (0.21, 25.48) | 2.95 (0.29, 29.72) | HQ+WM |  |  |  |  |  |  | 0.91 (0.40, 2.09) |
| 1.10 (0.02, 73.12) | 0.43 (0.02, 9.12) | 2.29 (0.11, 48.09) | 2.91 (0.07, 125.53) | 0.99 (0.04, 24.03) | FFDS+WM |  | NS |  |  |  | - |
| 2.00 (0.51, 7.80) | 0.79 (0.05, 12.55) | 4.18 (0.14, 121.20) | 5.31 (0.19, 145.00) | 1.80 (0.13, 25.19) | 1.82 (0.03, 96.83) | DZH+WM | NS |  |  |  | 0.32 (0.01, 8.23) |
| 1.10 (0.00, 346.61) | 0.43 (0.00, 62.88) | 2.29 (0.02, 331.94) | 2.91 (0.01, 675.08) | 0.99 (0.01, 156.81) | 1.00 (0.02, 51.21) | 0.55 (0.00, 147.21) | DH+WM |  |  |  | - |
| 1.33 (0.05,32.65) | 0.53 (0.08,3.39) | 2.78 (0.19,40.46) | 3.54 (0.26,47.64) | 1.20 (0.23,6.36) | 1.21 (0.04,36.63) | 0.67 (0.04, 12.05) | 1.21 (0.01, 221.32) | DSCX+WM |  |  | 0.73 (0.15, 3.48) |
| 0.47 (0.01,17.35) | 0.19 (0.02,2.26) | 0.99 (0.04,23.00) | 1.26 (0.09,17.14) | 0.43 (0.04,4.46) | 0.43 (0.01,19.04) | 0.24 (0.01, 6.64) | 0.43 (0.00, 101.69) | 0.36 (0.03, 4.95) | DS+WM |  | 3.05 (0.12, 76.39) |
| 0.26 (0.01,8.08) | **0.10 (0.01,0.96)** | 0.55 (0.03,10.41) | 0.70 (0.04,12.35) | 0.24 (0.03,1.87) | 0.24 (0.01,8.94) | 0.13 (0.01, 3.04) | 0.24 (0.00, 50.40) | 0.20 (0.02, 2.15) | 0.56 (0.03, 10.10) | CXQ+WM | 5.80 (0.68, 49.77) |
| 1.02 (0.06,17.60) | 0.40 (0.12,1.29) | 2.12 (0.22,20.14) | 2.70 (0.31,23.36) | 0.92 (0.40,2.09) | 0.93 (0.04,20.21) | 0.51 (0.04, 6.23) | 0.93 (0.01, 137.42) | 0.76 (0.18, 3.25) | 2.14 (0.24, 19.30) | 3.85 (0.58, 25.48) | WM |

The results of pairwise meta-analysis (upper right) and network meta-analysis (bottom left) for adverse reaction. Treatment estimates are shown as odds ratios (95% confidence intervals). The risk estimate is for the column-defining treatment compared to the row-defining treatment. Statistical significance is defined as 95% confidence intervals that do not overlap one. NS = adverse events in both groups were zero.WM = Western medicine, XST = Xueshuantong injection, YXY = Yinxingye injection, SXT = Shuxuetong injection, SK = Shenkang injection, HQ = Huangqi injection, FFDS = Fufangdanshen injection, DZH = Dengzhanhua injection, DH = Danhong injection, DSCX = Danshenchuanxiongqin injection, DS = Danshen injection, CXQ = Chuanxiongqin injection.

**Table S6. Network meta-analyses of serum creatinine**

| XST+WM |  |  |  |  |  |  |  |  |  |  |  |
| --- | --- | --- | --- | --- | --- | --- | --- | --- | --- | --- | --- |
| -23.34 (-77.38, 30.71) | YXY+WM |  |  |  |  |  |  |  |  |  |  |
| -15.88 (-75.59, 43.84) | 7.46 (-30.22, 45.14) | SXT+WM |  |  |  |  |  |  |  |  |  |
| -8.92 (-62.22, 44.38) | 14.41 (-17.91, 46.73) | 6.96 (-34.14, 48.05) | SK+WM |  |  |  |  |  |  |  |  |
| 4.54 (-48.44, 57.52) | 27.87 (-3.92, 59.67) | 20.42 (-20.27, 61.10) | 13.46 (-17.04, 43.96) | HQ+WM |  |  |  |  |  |  |  |
| -19.61 (-73.25, 34.03) | 3.72 (-23.32, 30.76) | -3.74 (-29.97, 22.50) | -10.69 (-42.33, 20.94) | -24.15 (-55.25, 6.94) | FFDS+WM |  |  |  |  |  |  |
| 1.20 (-40.45, 42.85) | 24.54 (-9.91, 58.98) | 17.08 (-25.71, 59.86) | 10.12 (-23.13, 43.38) | -3.34 (-36.08, 29.40) | 20.81 (-12.99, 54.61) | DZH |  |  |  |  |  |
| -16.98 (-73.49, 39.53) | 6.35 (-28.55, 41.26) | -1.10 (-40.00, 37.79) | -8.06 (-44.34, 28.22) | -21.52 (-57.33, 14.29) | 2.63 (-26.08, 31.35) | -18.18 (-56.36, 20.00) | DH+WM |  |  |  |  |
| -13.95 (-69.62, 41.72) | 9.39 (-26.71, 45.48) | 1.93 (-42.20, 46.06) | -5.03 (-39.99, 29.94) | -18.49 (-52.97, 15.99) | 5.66 (-29.82, 41.15) | -15.15 (-52.09, 21.78) | 3.03 (-36.65, 42.71) | DSCX+WM |  |  |  |
| 3.91 (-47.75 , 55.57) | 27.24 ( -2.30 , 56.78) | 19.79 (-19.16, 58.73) | 12.83 (-11.34, 37.00) | -0.63 (-28.17, 26.90) | 23.52 (-5.27, 52.31) | 2.71 (-27.85, 33.27) | 20.89 (-12.94, 54.72) | 17.86 (-14.55, 50.27) | DS+WM |  |  |
| -10.19 (-61.68, 41.30) | 13.15 (-16.09, 42.39) | 5.69 (-33.03, 44.41) | -1.27 (-29.10, 26.56) | -14.73 (-41.94, 12.49) | 9.42 (-19.06, 37.90) | -11.39 (-41.66, 18.88) | 6.79 (-26.77 , 40.36) | 3.76 (-23.47, 30.99) | -14.10 (-38.64, 10.45) | CXQ+WM |  |
| -26.18 (-74.74, 22.39) | -2.84 (-26.56, 20.88) | -10.30 (-45.04, 24.44) | -17.26 (-39.21, 4.70) | -30.72 (-51.89, -9.55) | -6.56 (-29.34, 16.21) | -27.38 (-52.35, -2.40) | -9.20 (-38.08, 19.69) | -12.23 (-39.44, 14.98) | -30.09 (-47.69, -12.48) | -15.99 (-33.09, 1.11) | WM |

The results of network meta-analysis for serum creatinine. Treatment estimates are shown as mean differences (95% confidence intervals). The risk estimate is for the column-defining treatment compared to the row-defining treatment. Statistical significance is defined as 95% confidence intervals that do not overlap zero. WM = Western medicine, XST = Xueshuantong injection, YXY = Yinxingye injection, SXT = Shuxuetong injection, SK = Shenkang injection, HQ = Huangqi injection, FFDS = Fufangdanshen injection, DZH = Dengzhanhua injection, DH = Danhong injection, DSCX = Danshenchuanxiongqin injection, DS = Danshen injection, CXQ = Chuanxiongqin injection.

**Table S7. Inconsistency and heterogeneity test of network meta-analyses**

|  | **Inconsistency** | | **Heterogeneity** | |
| --- | --- | --- | --- | --- |
|  | Ratio of consistent loop | *P* value | *I*^2^ | *P* value |
| Complete remission | 6/7 (85.71%) | 0.24 | 0% | 0.89 |
| Total remission | 7/7 (100.0%) | 0.19 | 0% | 0.99 |
| Serum albumin | 9/11 (81.81%) | 0.05 | 71.4% | <0.01 |
| Urinary protein excretion | 7/11 (63.64%) | 0.01 | 93.8% | <0.01 |
| Total cholesterol | 2/5 (40.00%) | 0.01 | 85.6% | <0.01 |
| Triglyceride | 1/3 (33.33%) | 0.12 | 97.7% | <0.01 |
| Serum creatinine | 3/4 (75.00%) | 0.38 | 95.9% | <0.01 |
| Adverse reaction | 2/2 (100%) | 0.80 | 0% | 0.98 |
